# The Role of Migratory Birds in the Dissemination of Antimicrobial Resistance: A One Health Perspective

**DOI:** 10.3390/vetsci13080782

**Published:** 2026-08-04

**Authors:** Ahmad Ali, Mohammad Adil, Bilal Ahmad, Muhammad Ilyas, Rakhshanda Rani, Uzair Alam, He Hongsu, Zhang Hui, Sun Zhihua

**Affiliations:** State International Joint Research Center for Animal Health Breeding, College of Animal Science and Technology, Shihezi University, Shihezi 832003, China; ahmadalisci1020@stu.shzu.edu.cn (A.A.); adilswabi15@gmail.com (M.A.); bilalsw33@gmail.com (B.A.); milyashussain2@gmail.com (M.I.); ranirakhshanda@yahoo.com (R.R.); uzairawkum13@gmail.com (U.A.); hongsuhe12@gmail.com (H.H.)

**Keywords:** antimicrobial resistance, migratory birds, antimicrobial resistance genes, One Health, wildlife reservoirs, environmental surveillance

## Abstract

Antimicrobial resistance (AMR) is a major One Health challenge driven by antimicrobial misuse in human medicine, veterinary practice, animal production, and polluted environments. Migratory birds move among wetlands, farms, wastewater-affected habitats, landfills, and coastal ecosystems and may acquire and redistribute antimicrobial-resistant bacteria (ARB) and antimicrobial resistance genes (ARGs) across ecological and political boundaries. This perspective synthesizes evidence on exposure sources, bacterial reservoirs, resistance determinants, cross-species interfaces, and surveillance priorities while explicitly distinguishing four claims: detection or carriage, persistence in individual birds, redistribution along migratory routes, and onward transmission to recipient hosts or environments. Published studies report multidrug-resistant *Escherichia coli*, *Klebsiella pneumoniae*, *Salmonella* spp., *Enterococcus* spp., and *Campylobacter* spp., with determinants including blaCTX-M, blaTEM, blaNDM, mcr, tet, sul, and qnr genes. The eight evidence groups summarized here constitute an illustrative, non-comprehensive selection; they are predominantly observational surveys or screenings, and none reconstructs a complete source–bird–destination–recipient transmission chain. Taxon-specific ecology modifies exposure: gulls and storks frequently exploit refuse and wastewater, waterfowl and shorebirds connect aquatic habitats, whereas passerines often reflect more local point-source contamination. Current evidence therefore supports migratory birds primarily as mobile sentinels and opportunistic carriers of anthropogenic AMR, while acknowledging possible natural or ancestral resistance in avian-associated microbiota. Future surveillance should combine longitudinal sampling, baseline cohorts such as pre-migratory nestlings, paired bird–water–soil–sediment sampling, whole-genome sequencing, plasmid profiling, telemetry, environmental DNA, wastewater-based epidemiology, and interoperable veterinary reporting. Practical mitigation requires antimicrobial stewardship, wastewater and landfill control, farm biosecurity, and coordinated veterinary, environmental, and public-health action.

## Simple Summary

Antimicrobial resistance makes infections harder to treat in people and animals. Migratory birds can travel long distances and may visit wetlands, farms, wastewater-affected areas, landfills, and coastal habitats. During these movements, they can acquire resistant bacteria and release them through feces. However, finding resistant bacteria in a bird proves carriage only; it does not by itself show long-term persistence, transport along a flyway, or transmission to livestock or people. This perspective evaluates these different evidence levels, explains how gulls, waterfowl, storks, pelicans, and passerines differ in exposure, and outlines practical veterinary surveillance and farm-biosecurity actions. Birds are best interpreted as mobile sentinels and occasional vectors within a wider system driven by antimicrobial use, waste management, and pollution. Coordinated sampling of birds, water, soil, livestock, and waste sites, combined with genome sequencing and movement data, can improve source attribution and early warning.

## 1. Introduction

**Figure 1:**
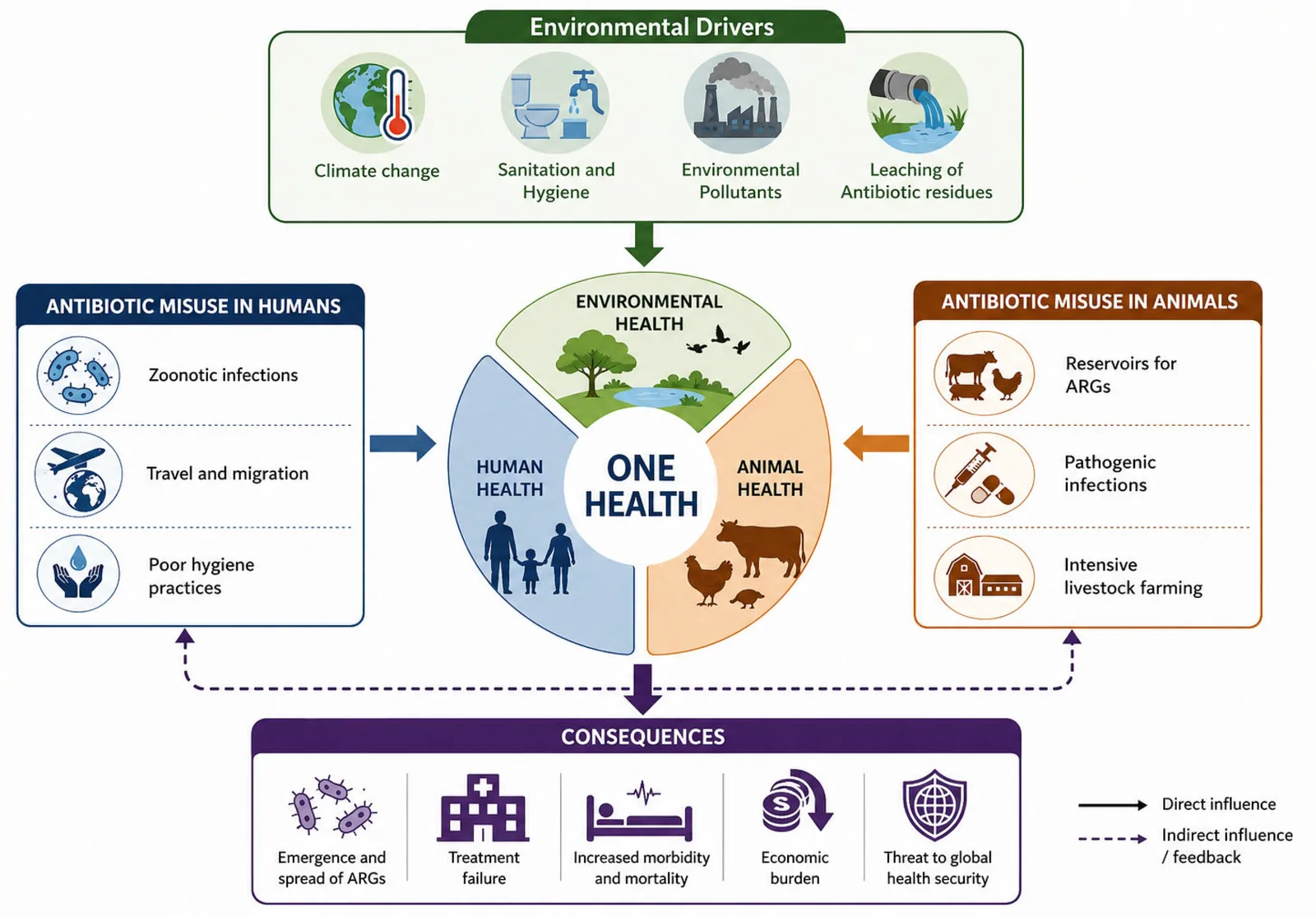
One Health framework for antimicrobial resistance (AMR) transmission among humans, animals, and the environment. Blue, orange, and green sectors represent human, animal, and environmental health, respectively; the purple panel summarizes downstream consequences. Solid arrows indicate direct influence or material flow, whereas purple dashed arrows indicate indirect influence or feedback. Environmental drivers act on the integrated system. ARGs, antimicrobial resistance genes. The diagram is conceptual and does not imply that every pathway has been demonstrated in a single transmission chain.

Antimicrobial resistance (AMR) is one of the most important threats to human, animal and environmental health. Resistance increases treatment failure, prolongs disease, raises medical costs and undermines progress in infectious disease control. Although clinical misuse of antibiotics is central to AMR emergence, resistance is also shaped by drug use in livestock, poultry, aquaculture and crop-associated systems, together with release of antibiotic residues, resistant bacteria and mobile genetic elements into the environment [1,2,3]. The One Health concept is therefore essential because AMR transmission occurs through linked human, animal and environmental pathways rather than within a single host population [4,5,6]. The One Health context of AMR transmission among humans, animals and the environment is summarized in Figure 1.

Environmental reservoirs are especially important because bacteria in water, sediment, manure-amended soil and biofilms can exchange resistance genes by conjugation, transformation and transduction. Residual antibiotics and heavy metals may select for resistant populations even outside hospitals or farms. Plasmids, integrons, transposons and bacteriophages can then move ARGs among commensal, environmental and pathogenic bacteria, producing a shared resistome that connects clinical, agricultural and wildlife interfaces [7,8,9,10,11,12].

Migratory birds are relevant to this system because many species travel long distances and repeatedly use habitats affected by human activity. Waterfowl, gulls, storks, shorebirds and passerines may feed in wetlands, fish ponds, poultry areas, refuse dumps and wastewater-impacted shorelines. During these movements, they can acquire ARB through water, sediment, food or contact with fecally contaminated substrates, and subsequently shed resistant organisms at distant stopover or breeding sites. For this reason, migratory birds are increasingly considered mobile sentinels and opportunistic vectors of environmental AMR [13,14,15,16,17].

The aim of this perspective is to evaluate how migratory birds contribute to AMR dissemination at the human–animal–environment interface. Unlike previous reviews that mainly catalog resistant organisms and genes or treat wildlife as a broad reservoir category, this article grades migratory-bird evidence according to causal strength: single-time-point carriage, persistence within identified birds, redistribution along flyways, and onward transmission to another host or environmental compartment. It also integrates taxon-specific ecology, natural or ancestral avian resistomes, genomic source-attribution criteria, and practical veterinary surveillance and biosecurity. This evidence-graded, implementation-oriented approach separates demonstrated findings from inferred pathways and links each evidence level to the study designs needed for stronger causal inference. The focus is relevant to veterinary and poultry health because migratory routes intersect livestock farms, poultry production zones, aquaculture ponds, and shared water bodies.

### Literature Identification and Scope

The evidence base was assembled through a targeted narrative approach rather than a systematic-review protocol. Studies were identified using combinations of the terms “migratory bird”, “wild bird”, “antimicrobial resistance”, “resistant bacteria”, “resistance gene”, “ESBL”, “One Health”, “flyway”, “wastewater”, “landfill”, “livestock”, “plasmid”, and “whole-genome sequencing”, together with backward screening of relevant review and primary-study reference lists. Peer-reviewed studies were prioritized when they reported AMR organisms or determinants in migratory or free-living birds, compared avian findings with environmental or domestic-animal interfaces, or informed persistence, movement, source attribution, or surveillance. Table 1 is an illustrative, non-comprehensive, purposive selection of eight evidence groups chosen to span geographical regions, avian taxa, study designs, and levels of inference; it is not intended as a prevalence meta-analysis or exhaustive inventory.

## 2. Sources of Exposure of Migratory Birds to ARB and ARGs

Human activities create multiple exposure points for migratory birds. Hospitals, community sewage, pharmaceutical residues and inadequate waste management release antibiotics, ARB and ARGs into wastewater networks and receiving waters. Healthcare waste may also enter open drains or poorly treated effluent streams, creating hotspots where clinically relevant bacteria and genes can persist before moving into rivers, lakes and wetlands used by birds [18,19,20].

**Figure 2:**
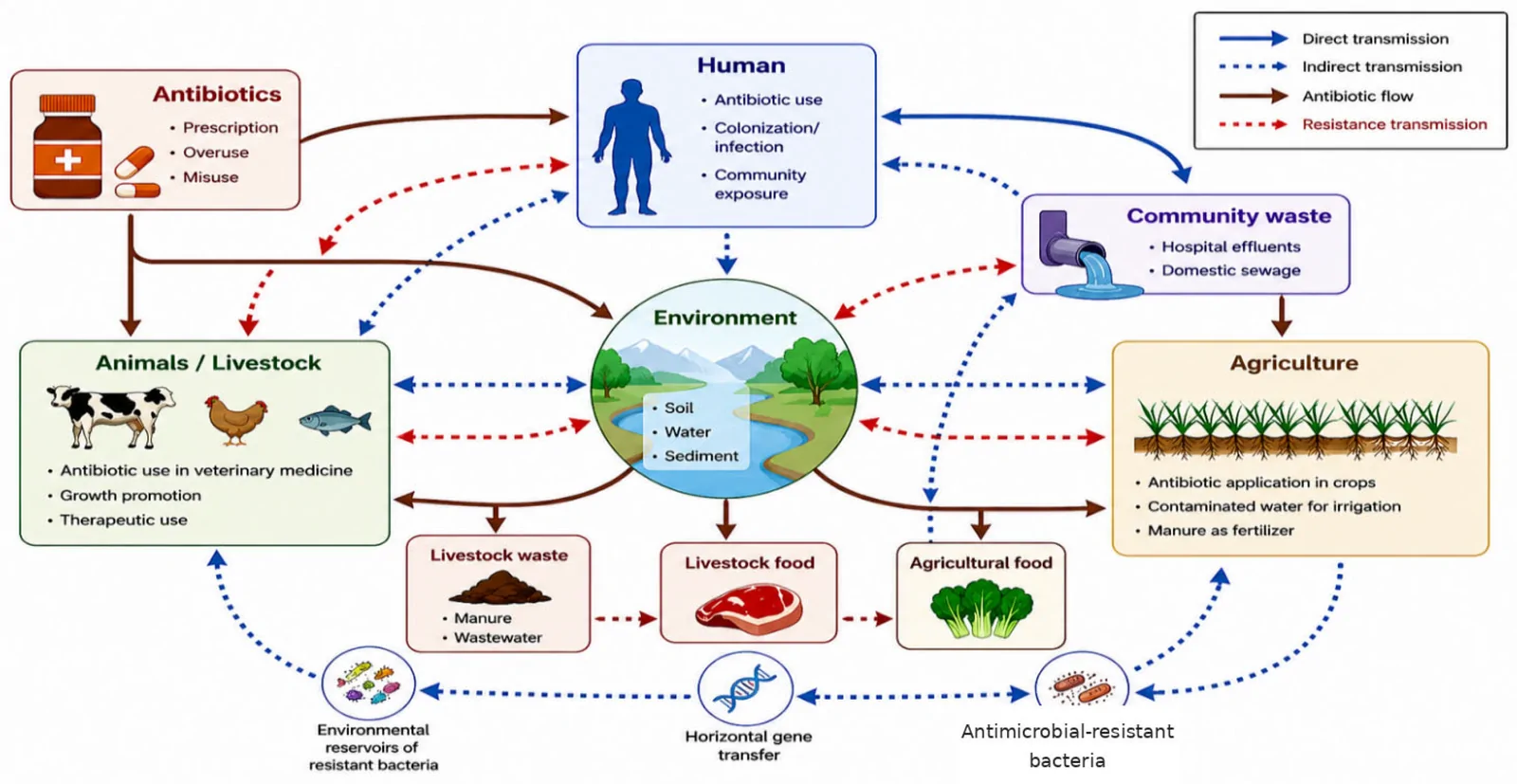
Conceptual pathways linking antimicrobial use, resistant bacteria, resistance determinants, and food-production systems. Blue solid arrows denote direct transmission, blue dashed arrows indirect transmission, brown solid arrows antimicrobial flow, and red dashed arrows resistance transmission. Boxes represent humans, livestock, community waste, agriculture, food products, and environmental compartments (soil, water, and sediment); the lower icons indicate environmental reservoirs, horizontal gene transfer, and antimicrobial-resistant bacteria. Bidirectional arrows indicate possible exchange, not proven directionality in an individual study.

Animal production is another major exposure route. Antibiotics are used therapeutically, prophylactically and, in some regions, for growth promotion in livestock, poultry and aquaculture. These practices select resistant bacteria in animal microbiota. Manure, litter, farm runoff and pond effluents can then spread ARB and ARGs into soil and water. Birds feeding in poultry yards, paddy fields, fish ponds, irrigated crops or livestock-adjacent wetlands may acquire resistant *Escherichia coli*, *Salmonella* spp., *Enterococcus* spp. and other organisms through ingestion or environmental contact [21,22,23,24]. Major routes linking human, animal and agricultural systems are shown in Figure 2.

Wastewater-treatment plants, landfills, sewage lagoons and polluted urban shorelines act as mixing environments where human, animal and environmental bacteria co-occur. Such sites promote genetic exchange because bacterial density is high and residues of antibiotics, disinfectants and metals may maintain selection pressure. Biofilms and sediment–water interfaces are particularly important because they can retain extracellular DNA and facilitate horizontal gene transfer. Migratory birds that feed or rest in these habitats can therefore become exposed to diverse AMR determinants before redistributing them through fecal shedding [25,26,27,28,29].

## 3. Environmental and Cross-Species Interfaces

**Figure 3:**
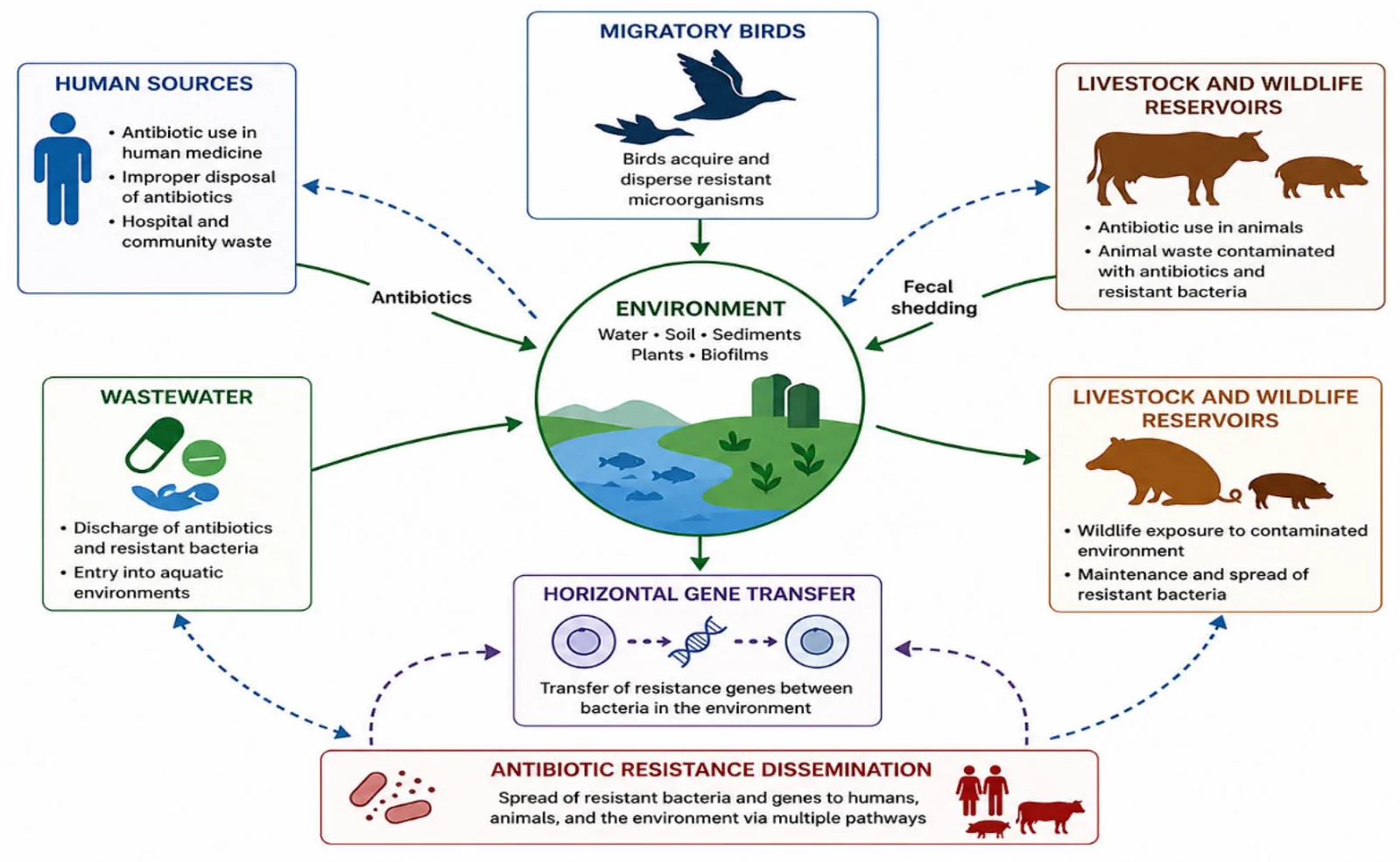
Mechanistic One Health pathways through which human sources, wastewater, migratory birds, livestock, wildlife, and environmental compartments may contribute to AMR dissemination. Green solid arrows indicate direct release, exposure, or fecal shedding; blue dashed arrows indicate indirect or bidirectional ecological connections; purple dashed arrows indicate pathways associated with horizontal gene transfer. The red lower panel represents the combined outcome of resistant bacteria and gene dissemination. These are plausible or inferred pathways unless supported by temporally ordered, strain- or plasmid-level evidence.

Migratory birds occupy shared landscapes where wildlife, livestock, domestic birds, and people use overlapping water and food resources. The central epidemiological question is therefore not whether exchange is biologically possible, but whether its direction and recipient can be demonstrated. Most available studies rely on cross-sectional detection and consequently support exposure or ecological connectivity rather than source status or transmission. Inference is stronger when bird, environmental, livestock, and human samples are collected in the same time window and compared at strain, plasmid, and mobile-element levels [30,31,32,33]. Figure 3 summarizes the mechanistic pathways that require testing.

Comparisons between highly impacted and lower-impact sites repeatedly associate human proximity with greater AMR carriage in birds. Such exposure gradients are informative because they provide an internal ecological contrast, and culture-based findings are increasingly supported by molecular characterization. Nevertheless, species composition, age, diet, season, sample type, antimicrobial panel, and laboratory method can confound site comparisons. Detection of the same phenotype or ARG in birds, surface water, or poultry is compatible with a shared source, but without temporal ordering and high-resolution genomic concordance, it does not establish transfer between compartments [25,34,35].

These limitations shift the management emphasis from blaming wildlife movement to reducing measurable selection and release pressures. Bird-associated findings should be interpreted as signals that identify interfaces for paired investigation, while intervention should focus on antimicrobial stewardship, waste treatment, runoff control, and farm biosecurity. This analytical distinction avoids repeating source descriptions and clarifies which observations support surveillance, ecological association, or causal transmission [36,37,38,39].

## 4. Migratory Birds as Reservoirs of ARB and ARGs

A growing number of studies have isolated ARB from free-living and migratory birds. *Escherichia coli* is the most frequently investigated indicator organism, but *Klebsiella pneumoniae*, *Salmonella* spp., *Enterococcus* spp. and *Campylobacter* spp. are also important because of their veterinary and zoonotic relevance. In China, migratory wild birds have been reported to carry multidrug-resistant *E. coli*, including ESBL-producing strains and plasmid-associated ARGs. Studies from river basins and multiple habitat sites further show that migratory birds may harbor virulence-associated and human disease-associated bacterial taxa, reinforcing their value for AMR monitoring [40,41,42].

European studies demonstrate similar patterns. Urban birds, seagulls, storks and pelicans have been used as indicators of environmental AMR, with reports of MDR *E. coli*, CTX-M-producing isolates and clinically relevant clones. Detection of ESBL-producing *E. coli* ST131-related lineages in urban seagulls is particularly concerning because such clones are also important in human infections. However, the presence of these clones in birds does not by itself prove bird-to-human transmission; it more likely indicates exposure to anthropogenic sources where human-associated bacteria are present [32,43,44].

Evidence from South Asia, the Middle East and Africa shows that migratory birds may carry MDR *Enterococcus* spp., *Salmonella* spp., *Vibrio* spp. and ESBL-producing Gram-negative bacteria in areas where wetlands, poultry operations and human settlements overlap. A systematic review of wild birds as reservoirs of antimicrobial-resistant *E. coli* supports the conclusion that prevalence varies widely by host species, sampling site and method, but that human-impacted environments repeatedly show higher risk [15,34,35].

**Figure 4:**
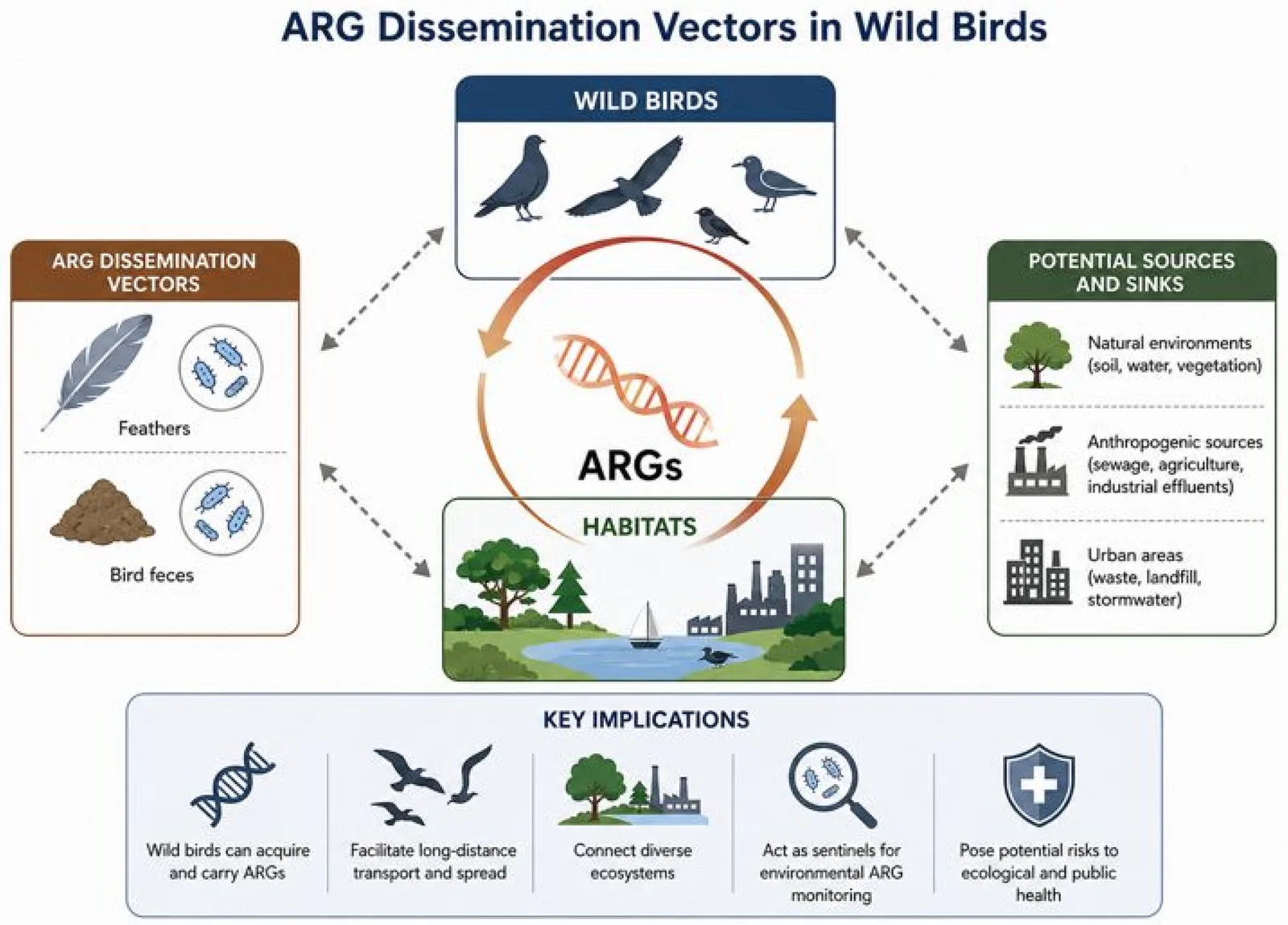
Potential cycling of antimicrobial resistance genes (ARGs) among wild birds and surrounding habitats. The orange circular arrows indicate possible acquisition, carriage, fecal release, and environmental reacquisition. Gray dashed arrows connect birds with dissemination vectors (feathers and feces) and with natural, anthropogenic, and urban sources or sinks. The lower panel summarizes surveillance and ecological implications. The figure illustrates hypotheses and monitoring targets; it does not by itself demonstrate long-distance transport or onward transmission.

The main ARGs reported from migratory or free-living birds include beta-lactamase genes such as blaCTX-M, blaTEM and blaNDM; colistin resistance genes such as mcr variants; tetracycline resistance genes including tet(A), tet(B), tet(M) and tet(X4); sulfonamide resistance genes such as sul1 and sul2; and quinolone resistance genes such as qnr. Phenotypic resistance to last-resort drugs in wild bird isolates is of special concern because it suggests that wildlife can encounter resistance determinants usually associated with intensive clinical or agricultural antibiotic pressure [27,45,46]. Dissemination from birds to surrounding habitats through fecal deposition is illustrated in Figure 4.

### 4.1. Taxon-Specific Ecology and AMR Exposure

Avian taxa differ in diet, habitat use, gut physiology, flock structure, and migration strategy and should not be treated as a single epidemiological unit. Gulls frequently move among wastewater-treatment plants, landfills, urban shorelines, and communal roosts; this repeated contact explains their value as indicators of human-associated *Escherichia coli* and CTX-M lineages but also creates substantial opportunity for local reacquisition [30,44]. White storks forage in refuse and agricultural landscapes and showed higher multidrug-resistant *E. coli* levels than sympatric seagulls in Central Spain, illustrating that species-specific foraging can be more informative than a simple migratory-versus-resident classification [32]. Waterfowl and shorebirds ingest surface water and sediment and can connect wetlands, rice fields, aquaculture ponds, and poultry areas, whereas passerines usually have smaller foraging ranges and may be more informative for local contamination around farms, gardens, or settlements [36,37]. Pelicans and other piscivorous birds may have different exposure profiles because their diet is less directly linked to refuse; a study reporting generally high antimicrobial susceptibility with occasional CTX-M-15-producing *E. coli* in free-living pelicans illustrates that clinically important isolates can occur even when overall resistance is low [43]. Surveillance should therefore stratify analyses by species, age, migratory status, feeding guild, habitat, and degree of human association rather than pooling all wild birds.

### 4.2. Evidence Levels for Carriage, Persistence, Flyway Redistribution, and Onward Transmission

Among the eight illustrative evidence groups in Table 1, seven are primary observational surveys or screenings and one synthesizes polar evidence; none follows marked birds across migration while simultaneously comparing matched source, destination, and recipient isolates. Detection by culture, PCR, or metagenomics at one time point supports carriage at sampling only. Persistence requires repeated recovery from the same individually identified bird, ideally before and after movement, with strain-level concordance. Migratory redistribution requires telemetry, banding, or repeated recapture that links the same bird or cohort to sequential sites together with high-resolution whole-genome and plasmid comparison. Onward transmission requires temporally ordered recovery of a genomically near-identical organism or mobile element from a plausible donor bird and a recipient animal, human, or environmental compartment, supported by exposure data, paired controls, and alternative-source assessment. Shared ARGs alone are insufficient because common genes may occur in unrelated strains and in ancestral environmental resistomes. Accordingly, terms such as reservoir, vector, and transmission should be qualified according to the evidence level actually demonstrated.

Polar and remote regions show that AMR is not restricted to densely populated areas, but detection in such settings does not uniquely indicate recent anthropogenic imports. Four categories should be distinguished. Intrinsic resistance is an inherent, usually chromosomally encoded property of a bacterial taxon, such as low permeability, constitutive efflux, or absence of a drug target. Ancient environmental ARGs are resistance determinants that predate modern clinical antimicrobial use and form part of long-standing environmental resistomes. Acquired but locally selected resistance results when horizontally acquired determinants are maintained or enriched by local antimicrobials, metals, disinfectants, or ecological competition. Recently introduced anthropogenic resistance refers to clinically or agriculturally associated strains, plasmids, or genes newly delivered through people, livestock, waste, research activity, marine connectivity, or bird movement. Foundational primary studies demonstrate both the deep evolutionary history and broad environmental distribution of resistance determinants [47,48,49]. Therefore, an Antarctic detection alone cannot identify which category is present. Distinguishing among these alternatives requires minimally impacted baseline sites, functional expression testing, genomic-context analysis, temporal sampling, and phylogeographic reconstruction [50,51,52].

Once colonization occurs, migratory birds may function as mobile ecological carriers, but the duration and direction of carriage remain uncertain and may depend on host species, gut microbiota, diet, stress, migration stage, and repeated exposure. At a contaminated site, a positive bird may have arrived already colonized or may have acquired the ARB locally; cross-sectional sampling cannot distinguish these alternatives. Whole-genome and global-database analyses show that wildlife *E. coli* can carry clinically relevant ARGs, but source attribution requires more than detection of a shared gene. It requires strain-level genomic relatedness, plasmid and mobile-element comparison, temporally ordered sampling, and ecological context [12,17,53].

### 4.3. Strengths, Limitations, and Geographical Coverage of the Evidence

The evidence base has important strengths: AMR carriage has been reproduced across multiple avian taxa and regions; culture-based phenotypes are increasingly supported by PCR, whole-genome sequencing, and plasmid data; and some studies include habitat or host comparisons. However, most investigations are cross-sectional, use convenience samples, emphasize *Escherichia coli*, apply heterogeneous susceptibility panels and breakpoints, and lack individually marked birds, pre-migration baselines, matched environmental or recipient isolates, and temporal ordering. These limitations prevent reliable estimates of persistence and directionality and can inflate causal language. Geographical coverage is also uneven: Europe and Asia provide most of the detailed ecological and genomic evidence, whereas African flyways and the Americas remain comparatively underrepresented. The current literature is therefore strong for demonstrating carriage and exposure gradients, moderate for ecological connectivity, and weak for complete flyway redistribution or onward-transmission chains.

**Table 1:** Illustrative evidence groups reporting antimicrobial-resistant bacteria and resistance determinants in migratory or free-living birds.

Region/Bird Group	Study Design	Main Findings	Key Resistance Determinants	Likely Interface and Inference Supported	Source
China/migratory wild birds	Cross-sectional surveillance	MDR *Escherichia coli* and ESBL-producing isolates reported	blaCTX-M, blaTEM and plasmid-associated ARGs	Wetlands and human-impacted habitats; cross-sectional carriage only	[40]
China/multiple habitat sites	High-throughput screening	Human disease-associated pathogens and ARGs detected in migratory birds	Multiple ARG classes	Ten habitat sites; broad occurrence and site association, not individual movement	[42]
Bangladesh/migratory birds	Fecal and microbiological survey	MDR *Enterococcus* spp., *Salmonella* spp. and *Vibrio* spp. detected	Multidrug resistance profiles	Wetland–poultry–community interface; carriage only	[35]
Pakistan/Trimmu Barrage birds and poultry	Phylogenetic analysis	ESBL determinants detected in wild bird and chicken fecal microbiota	ESBL-associated genes	Wild bird–poultry–water interface; shared ESBL determinants suggest connectivity, but directionality is unresolved	[34]
Spain/urban white storks and seagulls	Comparative urban sampling	Higher MDR *Escherichia coli* in urban-associated birds	Beta-lactam and multidrug resistance	Urban foraging and waste exposure; comparative ecological association	[32]
United Kingdom/urban seagulls	Molecular epidemiology	ESBL-producing *E. coli* ST131-related subclones identified	CTX-M-associated ESBL profiles	Urban gull–human waste interface; clone detection suggests source overlap, not bird-to-human transmission	[44]
Italy/wild birds	Phenotypic resistance survey	Resistance to last-resort antimicrobials in Gram-negative isolates	Carbapenem, colistin and other last-resort resistance phenotypes	Wild bird–environment interface; phenotypic carriage only	[45]
Antarctica/polar birds and ecosystems	Microbiome and environmental review evidence	AMR detected in a relatively pristine region	ARGs linked with environmental and movement pathways	Remote ecosystem connectivity; long-distance introduction, local acquisition, and ancestral resistomes remain alternative explanations	[50,51,52]

## 5. Long-Distance Transmission Along Migratory Flyways

Migratory flyways connect breeding, stopover and wintering areas across continents. Birds can encounter AMR hotspots during stopovers and later shed resistant bacteria in distant wetlands, agricultural landscapes or coastal areas. Gulls and waterfowl are often highlighted because they forage in highly contaminated settings and move across regional or international boundaries. This movement creates the possibility of transboundary AMR redistribution, especially for plasmid-borne ARGs that can transfer between bacterial hosts [16,30,51].

Long-distance spread should not be interpreted as a simple linear pathway from bird to human. Instead, flyways interact with socioeconomic and environmental gradients, including antibiotic availability, livestock density, wastewater treatment, landfill management and climate-driven habitat change. Birds may acquire resistance in one region, lose it during migration, reacquire it at another hotspot or transfer plasmids within their gut microbiota. Therefore, flyway-level risk assessment requires repeated sampling at multiple points rather than one-time detection in a single location [8,46,53].

Climate change may further alter AMR transmission by changing migration timing, stopover duration, wetland availability and bird–human–livestock contact patterns. Warmer temperatures and hydrological disturbance may also affect persistence of antibiotics, resistant bacteria and ARGs in freshwater ecosystems. These changes make migratory birds valuable indicators of emerging ecological shifts in AMR distribution, but they also complicate attribution because movement patterns and exposure sources are changing simultaneously [10,54].

## 6. Migratory Birds as Sentinel Species for Environmental AMR

The sentinel value of migratory birds lies in their exposure to multiple environments over large spatial scales. Sampling feces, cloacal swabs, feathers, and associated water or sediment can reveal where ARB and ARGs are accumulating. Compared with livestock surveillance alone, wildlife sampling can identify contamination beyond farms and clinics, especially in wetlands, reservoirs, protected areas, and migratory stopovers. Interpretation should nevertheless be species- and site-specific because a gull using a landfill, a waterfowl feeding in sediment, and a passerine using a farmyard represent different exposure histories [14,15,33].

A positive isolate from a bird does not prove that birds are the main source of resistance at a site. Avian AMR data are most interpretable when bird samples are paired, on the same dates and at the same foraging locations, with water, soil, sediment, manure, livestock, and wastewater samples. Studies should also record host ecology, migration status, feeding behavior, and local antimicrobial-use information and should compare avian isolates with human, livestock, and environmental isolates by whole-genome and plasmid analysis. Without these data, studies may overestimate long-distance transport, overlook local acquisition, or misclassify prior resistance as exposure at the sampling site [36,37,38].

## 7. Surveillance and Future Research Priorities

Current surveillance has important design and geographic gaps. Many studies are cross-sectional, culture-dependent, and focused on *Escherichia coli*, while fewer investigate whole microbial communities, plasmids, bacteriophages, or temporal persistence. To address prior resistance, cohorts should be sampled before expected exposure and repeatedly thereafter. Nestlings sampled before their first migration can provide a practical microbial baseline, followed by sampling before departure, at stopovers where feasible, and after return. Individually marked or telemetry-tracked birds, repeated seasonal sampling, and matched comparison groups are needed to estimate acquisition, clearance, persistence, and reacquisition rather than merely prevalence. Coordinated multi-country studies should give particular priority to underrepresented African and American flyways so that conclusions are not driven mainly by European and Asian surveillance [55,56].

Genomic tools should become central to source attribution. Whole-genome sequencing can define sequence types, virulence factors, core-genome or single-nucleotide-polymorphism relatedness, resistance islands, and mobile elements. Plasmid investigation should include replicon typing and, where possible, long-read sequencing to resolve complete plasmid structures, insertion sequences, and the physical linkage of ARGs. Metagenomics can characterize the broader resistome, including unculturable bacteria and low-abundance genes, but gene detection should be separated from evidence of expression, viability, and host assignment. Authentic source attribution requires genomic similarity together with temporal ordering, movement data, and matched environmental sampling [12,57,58].

International data integration remains limited. Human, livestock, food-sector, and wildlife datasets often use different sampling frames, susceptibility panels, breakpoints, metadata, and definitions. Veterinary authorities should align wildlife modules with existing national systems and relevant international frameworks, including the World Organization for Animal Health (WOAH), the European Food Safety Authority (EFSA), FAO food-and-agriculture surveillance tools, and GLASS-compatible One Health reporting where appropriate. Harmonization should include shared definitions of multidrug resistance, ESBL and carbapenemase production; standardized host and site metadata; comparable denominators; external quality assurance; and timely deposition of sequence data [59,60,61,62].

A minimum standardized field protocol should record species, age class, sex where feasible, body condition, migratory status, band or telemetry identifier, coordinates, date, habitat, flock size, feeding guild, and proximity to farms, wastewater-treatment plants, landfills, and surface water. Fecal or cloacal samples should be paired with water, sediment or soil, feed, manure, livestock, and wastewater samples from the same site and sampling window. Laboratories should report culture media, antimicrobial panels, interpretive breakpoints, quality-control strains, sequencing depth, assembly metrics, ARG database and version, plasmid methods, and raw-read accessions. Repeated seasonal sampling and harmonized denominators are necessary for valid prevalence comparisons and trend analysis [14,55,57,61].

Future studies should also quantify environmental drivers. Measurements of antimicrobial residues, metals, nutrients, bacterial abundance, and ARGs in water, sediment, manure, and landfill leachate can strengthen inference about exposure sources. Models may then integrate bird movement, environmental contamination, farm density, antimicrobial use, and genomic similarity to estimate risk, but they require independent validation before policy or intervention use [9,19,29,56].

### 7.1. Veterinary Implementation and Farm Biosecurity

Veterinarians should lead or co-lead investigations at poultry–wildlife interfaces by defining case and sampling frames, collecting animal and environmental specimens, interpreting phenotypic susceptibility and whole-genome results, tracing farm movements, and communicating uncertainty. Veterinary diagnostic laboratories can connect wildlife isolates with livestock and food-chain databases, while field veterinarians can report unusual mortality, treatment failure, or spatial clusters and coordinate with wildlife, environmental, and public-health agencies. Public communication should avoid presenting wildlife as the primary cause when the evidence supports only environmental exposure or carriage; messages should emphasize controllable sources and pathways.

At farms near wetlands or migratory stopovers, practical biosecurity includes preventing wild-bird access to feed and drinking systems; draining, covering, or fencing unnecessary standing water; repairing leaks; protecting feed stores; cleaning and disinfecting equipment; separating clean and dirty traffic; managing manure and litter; promptly collecting and securely disposing of carcasses; discouraging scavenging at waste areas; and monitoring wells, surface water, sediment, and runoff. During high-risk migration periods, farms should intensify environmental sampling and review antimicrobial use, but indiscriminate wildlife culling should be avoided because it does not address the underlying selection pressure [21,22,23,24].

### 7.2. Emerging Surveillance Technologies

Artificial intelligence and machine-learning models can combine land use, antimicrobial sales or use, wastewater quality, weather, bird abundance, migration timing, and farm density to prioritize surveillance sites, although models must be externally validated and checked for geographic and sampling bias. Satellite or GPS tracking linked to whole-genome and plasmid data can identify high-risk migratory pathways and separate local exposure from plausible long-distance carriage. Environmental DNA offers non-invasive screening of water and sediment, but it does not necessarily identify the host, viability, or direction of transmission; positive signals therefore require confirmatory culture or targeted sequencing. Wastewater-based epidemiology can provide early warning near urban, livestock, and food-production interfaces. A tiered system combining these technologies with conventional microbiology and field epidemiology is more informative than any single method [56,57,61,63].

**Figure 5:**
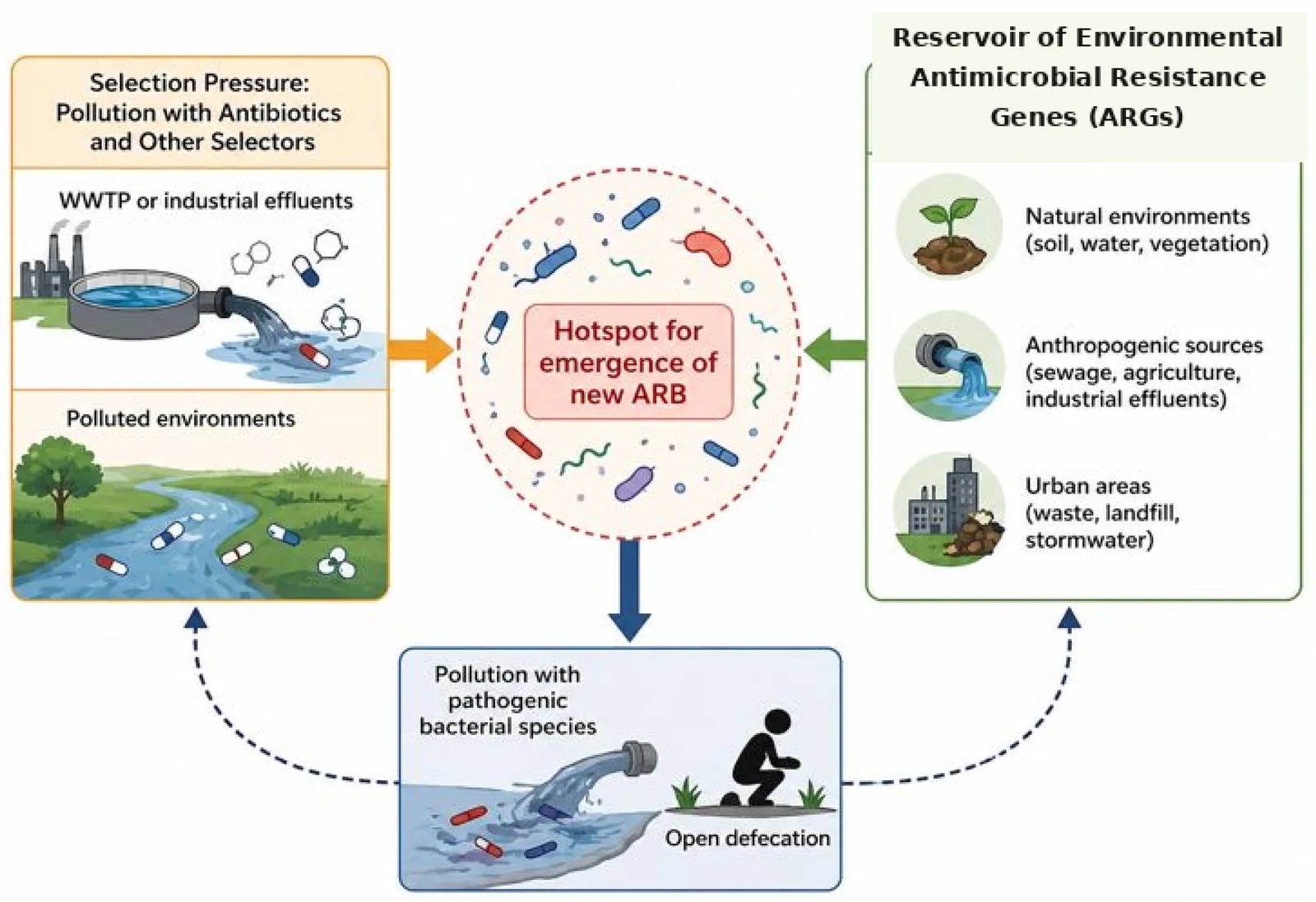
Environmental hotspots for the emergence and dissemination of antimicrobial-resistant bacteria (ARB). Orange and green solid arrows indicate contributions from selection pressure and environmental resistance-gene reservoirs to an AMR hotspot; the blue solid arrow indicates downstream contamination with pathogenic bacteria. Blue dashed arrows represent indirect feedback from contaminated receiving environments and open defecation to upstream source compartments. WWTP, wastewater-treatment plant; ARGs, antimicrobial resistance genes. The pathways are conceptual and include both demonstrated source pressures and inferred feedback routes.

Management should prioritize the sources that create AMR selection pressure: rational antimicrobial use in humans and animals, restrictions on unnecessary prophylaxis and growth promotion, farm biosecurity, safe manure management, effective wastewater treatment, landfill control, and protection of wetlands from contaminated inflows (Figure 5). Constructed wetlands and other nature-based barriers may reduce pathogen and ARG movement when carefully designed and monitored. Veterinary professionals are central to stewardship, surveillance at poultry–wildlife interfaces, implementation of farm controls, and communication among animal, human, wildlife, and environmental health sectors [21,22,23,24,28,29].

## 8. Conclusions

Migratory birds are important ecological participants in the One Health AMR system because they connect contaminated and relatively natural habitats across large geographic scales. Evidence robustly confirms carriage of multidrug-resistant bacteria and clinically relevant ARGs, but it does not yet establish persistence, flyway redistribution, or onward transmission as general phenomena. Cross-sectional detection cannot determine whether resistance was acquired before arrival or locally, and findings in remote regions may represent intrinsic, ancient, locally selected, or recently introduced resistance. Migratory birds should therefore be interpreted primarily as mobile sentinels and opportunistic carriers within systems driven by antimicrobial use, wastewater, agricultural runoff, manure, and landfill exposure.

The practical implication is a risk-based surveillance and control strategy. Veterinary and public-health authorities should prioritize sentinel sampling at wetland–poultry, wastewater, landfill, and aquaculture interfaces; collect paired bird, water, sediment, manure, livestock, and waste samples; and use whole-genome, plasmid, and movement data when high-risk clones or last-resort resistance determinants are detected. Farms near migratory stopovers should protect feed and drinking water, manage standing water and waste, and intensify environmental monitoring during peak migration. Harmonized reporting and expanded surveillance in Africa and the Americas are needed to correct geographic bias. A positive bird sample should trigger source investigation and targeted source control, not automatic attribution to wildlife or indiscriminate culling. This evidence-graded framework converts uncertainty into specific research, veterinary, and biosecurity actions.

## Data Availability

The original contributions presented in this study are included in the article. Further inquiries can be directed to the corresponding authors.
